# Study Progress on Inorganic Fibers from Industry Solid Wastes and the Key Factors Determining Their Characteristics

**DOI:** 10.3390/ma15207256

**Published:** 2022-10-17

**Authors:** Jincai Zhang, Xing Xu, Fangqin Cheng, Seeram Ramakrishna

**Affiliations:** 1 Institute of Resource and Environment Engineering, Shanxi University, No. 92 Wucheng Road, Taiyuan 030006, China; 2 Department of Mechanical Engineering, National University of Singapore, Singapore 117574, Singapore

**Keywords:** inorganic fiber, chemical composition, melt structure, viscosity, solid wastes

## Abstract

Compared to basalt and glass fibers, the production of inorganic fiber from industry solid wastes is an effective method to not only save natural resources but also recycle waste resources. Because the preparation of the fibers requires high temperature treatment, the production process is associated with high energy consumption and high carbon emissions. How to resolve these problems is a current research challenge in this field. Herein, we reviewed the study progress on these fibers and further discussed the key factors determining their characteristics, including chemical composition, melt structure, and viscosity of melt. In production, the matching of solid waste blends containing enough total content of SiO_2_ and Al_2_O_3_, and a suitable amount of MgO and CaO, is beneficial to the structure control of the melt. The study found that the melt consisted of Q^2^ and Q^3^; and that Q^3^ content more than Q^2^ was more suitable for fiber production and its performance improvement. Such a melt structure can be achieved by controlling the degree of depolymerization and the temperature. New ultrasonic technology can shorten the homogenization time; its application is hoped to save energy and reduce carbon emissions. These conclusions will offer important guidance for the development of inorganic fibers from industry solid wastes in the future.

## 1. Introduction

Basalt continuous fibers (BSFs) and glass fibers are two types of important engineering materials used in various industries [1,2]. As short fibers, the mineral wool fibers are often processed into felt, boards, and thermal insulation material and are widely used in building insulation. The larger-scale production of these fibers requires the extraction of large quantities of natural minerals, which not only destroys the ecological environment, but also brings inconvenience to people’s lives. Finding cheap alternative materials to produce such fibers is an effective way to solve the current dilemma. Over 40 years, the rapid development of industry has produced ten billion tons of solid wastes in China, including coal gangue, fly ash, various tailings, metallurgical waste, desulfurized gypsum, carbide slag, hazardous solid waste, etc. The recycling of these resources has become a very urgent problem faced by humans and the environment. Considering the chemical composition of some industry solid wastes is similar to natural minerals, such as SiO_2_, Al_2_O_3_, CaO, MgO, Fe_2_O_3_, K_2_O, and Na_2_O, it is possible to try to make fibers from them. As early as the 1970s, the Soviet Union reported the short fibers made from fly ash and clay showed excellent fire resistance [3], which attracted extensive attention. At the same time, Japan, the United States and some European countries also carried out many studies on fly ash-based mineral fiber [4]. In China, related research started in the 1990s [5], after which various solid waste slags, such as blast furnace slag (BFS), tailings, fly ash, and steel slag, etc, were tried to prepare mineral fiber [6]. After nearly 30 years of research and development, slag-based mineral fibers have undergone rapid development. At present, these mineral fibers have been widely produced and used in China. In contrast, studies on the continuous fibers produced from solid wastes are in the nascent stage. Recent study results [7,8,9,10] were very inspiring; an inorganic continuous fiber from fly ash and magnesium slag had a tensile strength of 903 MPa and its tensile strength retention rate (TSRR) was 68.8% after 400 °C treatment. The TSRR was slightly higher than continuous basalt fiber (67.76%) at the same temperature treatment [11]. Further studies [12,13] indicated the continuous fibers from fly ash and other slags exhibited a tensile strength of 1823 MPa. Such rapid study progress suggests that it is hopeful that these continuous fibers could partially replace BCFs or glass fibers in the future. This is of great significance to the whole fiber industry and the recycling of solid waste resources.

Regardless of which fibers, the production process needs to melt raw material into uniform melt at a high temperature, and then make the fiber. The whole production process includes the following three steps: selection of raw materials, melting–homogenization and fiber-forming. The latter two steps are accompanied by high energy consumption and high carbon emissions. How to resolve these problems? What is the current research status? Up until now, there has been a lack of comprehensive understanding of inorganic fibers produced from various solid wastes, even for BCFs. Herein, we summarize the research progress of these fibers and review the key factors affecting their performance. It is essential to advance the level of research and promote the development of inorganic fibers produced from solid waste resources.

## 2. The Production Process of Inorganic Fibers from Solid Wastes

**Figure 1:**
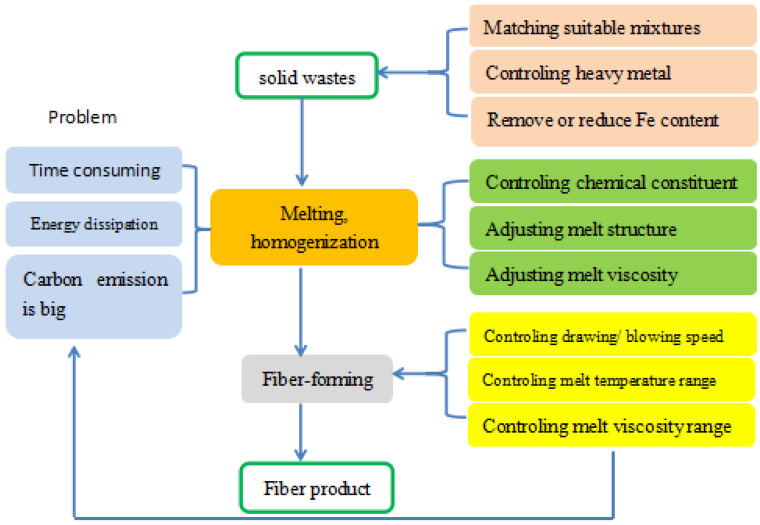
Production steps and control factors of the slag-based fibers.

Figure 1 shows the production steps of the above inorganic fibers. Firstly, raw materials need to be screened out and matched to meet the requirements. Secondly, these solid waste blends should be treated at high temperature to completely melt and achieve a uniform and stable vitreous state. This process is known as “melting–homogenization”. Finally, the melt is converted into fibers, named the fiber-forming stage.

### 2.1. Selection of Solid Wastes for the Preparation of the Inorganic Fibers

Because of the difference in the composition of various solid wastes, it is necessary to screen out suitable solid wastes to produce fibers. For environmental protection requirements and to avoid human health hazards, the content of heavy metals [14,15,16] in solid waste needs to be controlled. Currently in China, the national standards (NSs) for these fibers from solid wastes are still not formulated. In practice, the reference NS is the Identification standards for hazardous wastes—identification for extraction toxicity (ISHWIFET) (GB 5085.3-2007). Limits for heavy metal content are set in this NS. Another NS is Limits of radionuclides in building materials (LRBM) (GB6566-2010), which sets specific limits for radioactive elements in building materials, such as Ra-226, Th-232, and K-40. The production of fibers from solid wastes requires mixing several solid wastes to meet the chemical composition requirements. What chemical composition ensures high performance fiber production will be discussed in detail in Section 3.2.

### 2.2. Melting–Homogenization

In melting–homogenization, also named as vitrification, the aim is to convert the various crystalline and amorphous solid components into homogeneous glassy states at a high temperature condition [17]. This process is very important for the production of fibers for the following reasons. To obtain high-performance fiber products, the melt must meet the following three requirements at this stage: uniform chemical composition, reasonable melt structure and stable melt viscosity as well as avoiding any crystals inside the melt. In practice, the melting–homogenization consumes a lot of time, energy and produces a large amount of carbon emissions. Therefore, it is very necessary to develop a fast homogenization technique. Specifically, there are obvious differences in the production of mineral fibers and continuous fibers from solid wastes. When mineral fibers are produced, the fiber-forming temperature (T_ff_) is up to 1450–1550 °C due to the low viscosity requirement [18]. The melting–homogenization temperature (T_mh_) is very near the melting point; the hold time is 15–60 min [19,20]. In contrast, the fiber-forming viscosity (V_ff_) of continuous fibers is higher, and the T_ff_ is in the range 1200–1410 °C. The T_mh_ is over the T_ff_ 120–220 °C [8,10].The homogenization time usually is very long and up to 4 to 8 h [21]. To shorten the time and reduce energy consumption and carbon emissions, even in high-capacity factories, the general solution is to limit the capacity of a single device and increase the number of devices. In this way, energy and time are saved by reducing the amount of homogenization materials on a single device. However, this is only a way to save energy from the point of view of equipment configuration. Only by shortening the homogenization time can energy be saved and carbon emissions reduced. New technological breakthroughs are urgently needed in this area.

### 2.3. Fiber-Forming Process

**Figure 2:**
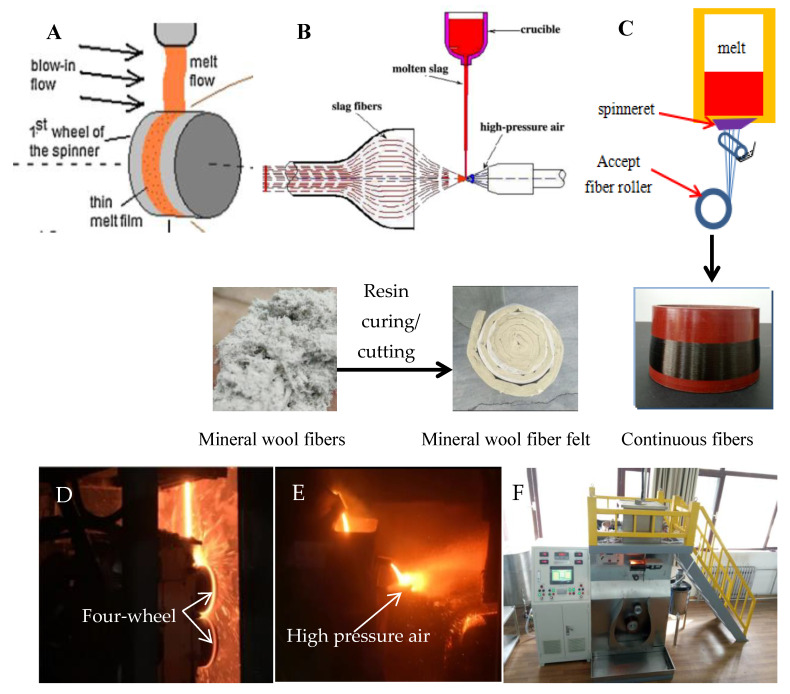
Schematic diagram of fiber production. (**A**) Rotating centrifugation (Chan et al., 2017). (**B**) Air blowing (Wang et al., 2010). (**C**) Spinneret drawing. (**D**) The production site of rotating centrifugation (Zhao et al., 2018). (**E**) The production site of air blowing. (**F**) The production site of continuous fibers.

Figure 2 indicates the fiber-forming process of both mineral and continuous fibers. The mineral fibers are manufactured by rotating centrifuge or high pressure air blowing (Figure 2A,B) [17,22]. The vitreous melt falls onto four wheels with a rotating speed of 2500–8500 rp/min [19,23] and is converted into mineral fibers by centrifugal shear forces; it is known as rotating centrifugation. Air blowing uses high-pressure air to blow the melt into short fibers. Then, the obtained fibers are cured by resin and cut into the required size of the products according to the needs of the application. The continuous fibers are prepared by spinneret drawing (Figure 2C). The high-temperature melt flows out from the spinneret and is instantaneously stretched into micron-sized fibers under the action of traction. The drawing speed varies from several meters to tens of meters per second depending on the requirement of fiber diameters. Figure 2D–F show the production sites and equipment [19].

## 3. The Key Factors Determining the Performances of the Fibers

### 3.1. Composition of Vitreous Melt

The transformation process from melt to fiber was finished in a very short time. In this process, the temperature dropped rapidly from T_ff_ to room temperature and the structure of the melt was instantaneously retained in the fibers. In other words, the structure of the melt predestined the structure of the fibers. Generally, the composition of vitreous melt can be roughly divided into three categories: network formers, network modifiers and amphoteric agents [24]. Network formers (Si^4+^, Ge^4+^ and Ti^4+^) endowed the glassy melt with fiber-forming ability by forming a network structure consisting of Si-O, Ge-O, and Ti-O bonds. The oxygen atoms that connect Si, Ge and Ti atoms are called bridging oxygen (BO) atoms. The network modifiers (Ca^2+^, Mg^2+^, Na^+^, K^+^, Fe^2+^, Cr^3+^, V^5+^, Ba^2+^, Sr^2+^, Ti^4+^) filled in the voids or edges of the network structure by valence electric attraction of non-bridge oxygen (NBO) atoms [25], which could control the polymerization degree of the network structure by depolymerizing the network structure, so that the melts had glass fluidity. The existence of Ti^4+^, as a network former or network modifier, depended on its coordination number. It could enter the silica network of a partially depolymerized silicate melt in an octahedral form. It also could participate in the network structure in a tetrahedral form. Al^3+^ and Fe^3+^, as amphoteric agents, had some complex effects on melt properties.

### 3.2. Chemical Composition of Fibers

The chemical composition of solid wastes is a very critical factor that determines the mineral and crystal compositions, and further determines the melting point and homogenization temperature and time of the mixture of various solid wastes. Chemical composition and temperature jointly determine the structure, viscosity of melt and fiber properties. The melts with homogeneous chemical composition and structure have similar properties, including viscosity, fluidity, surface tension and crystallization properties. This becomes the theoretical basis for the preparation of the fibers using solid wastes. As far as we know, the research into continuous fibers produced from solid wastes is just starting; there are a few related publications. Here, discussion includes the influence of chemical composition on the properties of BCFs. Theoretically, it still has important guiding significance for the research into continuous fiber production from solid wastes.

#### 3.2.1. Influence of Chemical Composition on the Properties of BCFs

**Figure 3:**
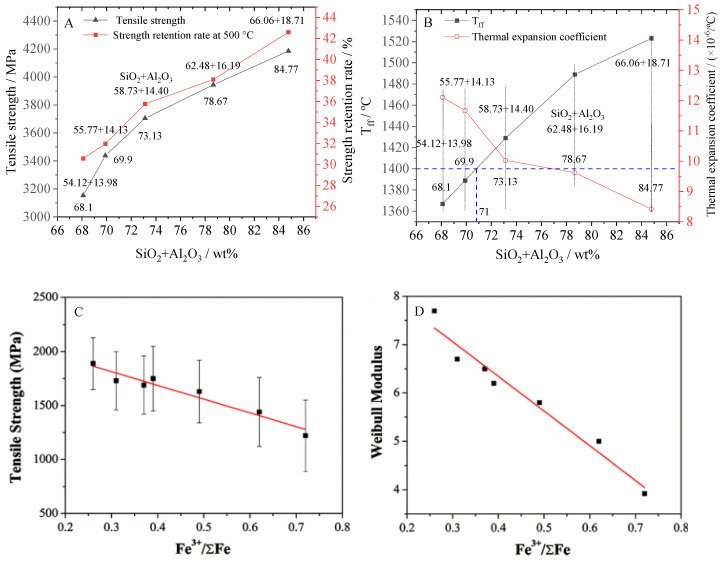
Effect of main chemical composition on the mechanical properties and thermal stability of the BCFs. (**A**) The effect of the total content of SiO_2_ and Al_2_O_3_ on the tensile strength of the BCFs and its strength retention rate at 500 °C. (**B**) The effect of the total content of SiO_2_ and Al_2_O_3_ on the T_ff_ and the thermal expansion coefficient of the BCFs; the data of (**A**,**B**) from (Liu et al., 2017). (**C**) The relationship curve between the Fe^3+^^/^ΣFe and tensile strength of single continuous fiber (Xing et al., 2019). (**D**) The relationship curve between the Fe^3+^^/^ΣFe and Weibull modulus of the single continuous fiber (Xing et al., 2019).

The main chemical compositions of BCFs are as follows: SiO_2_ > Al_2_O_3_ > CaO > MgO > Fe_2_O_3_. The mechanical performance of fibers mainly depends on the network structure consisting of network formers. SiO_4_ tetrahedron is the basic unit of network structure. Some Al atoms also participate in the network structure in the form of AlO_4_ tetrahedron. The available literatures [26,27,28,29,30] show the increase of SiO_2_ and Al_2_O_3_ total content can promote the development of network structure and improve the mechanical strength of fiber and thermal stability. Figure 3A shows that the tensile strength of the fibers and its strength retention rate at 500 °C increase with the total content of SiO_2_ and Al_2_O_3_ or any single component content. Figure 3B indicates the T_ff_ of the fibers also increases with the total content of SiO_2_ and Al_2_O_3_ or any single component content. However, the thermal expansion coefficient of the fibers decreases as the total content of SiO_2_ and Al_2_O_3_ or any single component content increases. The tensile strength of the fibers is up to 4185 MPa when the total content of SiO_2_ and Al_2_O_3_ is 84.77% (SiO_2_ + Al_2_O_3_:66.06% + 18.71%). However, during the actual production, the T_ff_ normally should not exceed 1400 °C because at high temperatures the bushing will creep, which will lead to deformation, reducing service life and seriously reducing the drawing efficiency [31].Therefore, the total content of SiO_2_ and Al_2_O_3_ in basalt should not be more than 71%, not the 78% as previously reported. Generally, SiO_2_ ≤ 51% and Al_2_O_3_ ≤ 20% is suitable, too high a content of Al_2_O_3_, which will increase the drawing temperature and melt viscosity, resulting in drawing difficulties and cost increases.

Iron ions exist in two valence states of Fe^3+^ and Fe^2+^. Fe^3+^/ΣFe increasing can reduce the tensile strength and Weibull modulus of the BCFs (Figure 3C,D) [32]. During the fiber-forming process, the high-temperature melt is in an oxidizing atmosphere, which will lead to the transformation of Fe^2+^ to Fe^3+^, and improve the Fe^3+^^/^ΣFe value, resulting in the decline of fiber quality. However, it is difficult to control the conversion of Fe^3+^ to Fe^2+^ in a reducing atmosphere; once an over-reduction occurs, Fe makes the platinum–rhodium plate toxic. Therefore, the content of iron oxide needs to be strictly controlled. It is suggested to remove the ferromagnetic components by magnetic separation of iron-containing raw slags before melting. Effective iron removal is a good choice to improve fiber quality and thermal stability [33]. Considering the cost of iron removal, it is more suitable to produce continuous fibers from waste slag without iron or as little iron as possible.

CaO and MgO belong to basic oxides, and they can replace each other to some extent. The increase of both contents can reduce the melting temperature of raw materials. Increasing the MgO content will increase the crystallization tendency of the melt. CaO can increase the surface tension of the melt, and the content of both should be controlled within an appropriate range. Other trace constituents also have various influences on melt and fiber. A small amount of TiO_2_ can improve the chemical stability of the fibers, melt surface tension and viscosity, which is conducive to fiber-forming. K_2_O and Na_2_O can effectively reduce the melting temperature of raw materials, and melt viscosity, expanding the range of drawing temperature. Meanwhile, it also reduces the chemical durability and thermal resistance of the fibers. Their contents and CaO and MgO components increase and favor the production of thick fibers. MnO can decrease the melting temperature and improve the chemical stability of the fiber. Cr_2_O_3_ can improve the chemical corrosion resistance of fibers and the surface tension of the melt; however, slag balls are easily generated in the drawing process, thus reducing fiber yield [34]. 

A small amount of ZrO_2_ can inhibit the deposition of Si and Al ions in the impregnated alkali solution, reduce the overflow of the corroded components, and improve the alkali resistance of the fiber [35]. In addition, those ions with a small ionic radius and strong polar ability (such as Li^+^, Be^2+^, Mg^2+^, Al^3+^, Ti^4+^ and Zr^4+^) can also improve the elastic modulus of the fibers. The order of influence of different oxides on the elastic modulus of fiber is as follows: CaO > MgO > B_2_O_3_ > Fe_2_O_3_ > Al_2_O_3_ [11].

In industry, it is customary to introduce an acidity coefficient (Mk) to represent the main chemical composition. Mk = (SiO_2_ + Al_2_O_3_)/(CaO + MgO) (mass ratio). High Mk value means the fiber has a developed network structure and excellent mechanical strength and thermal resistance. However, the fiber performance is poor. Continuous fibers pursue their own mechanical strength, so the Mk is usually high. An earlier study reported that the appropriate Mks for the production of continuous fibers were 2.0–2.35 [9]. A more recent study [36] showed the Mk range could be extended to 2.0–7.0, and at the same time it had an important effect on melt viscosity, density, and mechanical and crystallization properties of the fibers [37,38]. 

#### 3.2.2. Difference in Chemical Composition between Mineral Fibers and Continuous Fibers Produced from Solid Wastes

The mineral fibers from natural ores have a wide chemical composition (wt%): 38–55 SiO_2_, 3–20 Al_2_O_3_, 0–17 CaO, 1–24 MgO, 2–18 (FeO + Fe_2_O_3_), 0.3 MnO [39,40]. To obtain high-quality basalt mineral fibers, the chemical composition of raw materials is required to meet more stringent requirements (wt%): 41–50 SiO_2_, 19–30 Al_2_O_3_, 12–22 CaO, 0.5–9 MgO [41]. Table 1 indicates the corresponding data of mineral fibers from solid wastes. The majority of these mineral fibers need to be prepared at the high-temperature of 1450 to 1600 °C except for the fiber from MSS. The mean diameter of fibers is less than 7 µm except for the fibers from CSCD. The fibers produced from BFS have the highest tensile strength, up to 2579 MPa, due to the higher total content of SiO_2_ and Al_2_O_3_ (62.80%) and the highest content of Al_2_O_3_ (26.70%). The second highest tensile strength is held by the mineral fibers from CSCD with a tensile strength up to 1806 MPa. The series fibers from ferronickel slag have a small total content of SiO_2_ and Al_2_O_3_ (51.58% to 54.63%); however, their tensile strength is still high, from 1724 to 2114 MPa, due to the effect of B_2_O_3_. Table 1 indicates the Mk range of mineral fibers is from 1.20 to 3.07. The tensile strength of the fibers with high Mk is obviously larger, for example, the fibers from BFS and CSCD have a Mk of 1.99 and 3.07, and their tensile strengths are 2579 MPa and 1806 MPa, respectively. However, the T_ff_ is up to 1600 °C when the Mk is ≥1.80 and this is too high if considering the energy consumption and service life of a machine. The suitable Mk range should be 1.20 to 1.80.

Table 2 lists the corresponding data of continuous fiber from solid wastes. The T_ff_ of these fibers is in the range of 1200 to 1410 °C. The mean diameter of the fibers is from 9.11 to 61.1μm. The strength of the fine fiber is obviously higher than that of thick fiber, such as Faf2, Faf3, VF1,VF3, and VF6; their diameters are less than 15 µm and tensile strengths are more than other fibers. Compared with BCFs, the tensile strength of these continuous fibers from solid wastes do not always keep a positive growth trend as the total content of SiO_2_ and Al_2_O_3_ increase. For example, the tensile strength of VF6 (1571 MPa) is more than that of VF1 (1268 MPa), but its total content of SiO_2_ and Al_2_O_3_ (52.3%) is less than VF1 (66.8%). Faf3 and Faf 1 are similar cases. However, the improvement effect of Al_2_O_3_ on the tensile strength of the continuous fibers is obvious. VF1 and VF3, with their total content of SiO_2_ and Al_2_O_3_ (66.8% and 67.5%), are very closed. Their tensile strengths are greatly different, the former (1268 MPa) is far less than the latter (1823 MPa) due to the 4.8% of difference in the Al_2_O_3_ content. Table 2 shows the Mk of the continuous fibers are in the range of 1.48 to 8.04. When the Mk is too high, the melt has difficulty forming continuous fibers due to the effect of many factors, such as high T_ff_ and high V_ff_. Faf1 has the Mk of 8.04, but it is incapable of fiber-forming at a winding rate greater than 300 rpm [12]. Comparing the data in Table 2, it can be found that continuous fibers have a good tensile strength when the Mk is in the range of 1.48 to 4.59 and fiber diameters are less than 15 µm.

### 3.3. Fiber and Melt Structure

As is known, the performances of fibers depend on their structure. The fibers’ structure depends on the structure of the melt, determined by the chemical composition and temperature. The network structure consists of SiO_4_ tetrahedron via BO, a key factor that makes the melt transfer into fiber under traction or centrifugal forces. It is why the raw material is required to contain enough SiO_2_. For fiber produced from solid wastes, Al_2_O_3_ content in raw form is often second only to SiO_2_. It can participate in the network structure composed of Si-O on the condition of Al_2_O_3_ ≤ MO + M_2_O (mol%) (M is alkali and alkaline earth metal) [46,47]. When Al_2_O_3_ > MO + M_2_O, it will occupy 5, 6, or even higher coordination sites, weakening the network modification behavior due to lacking charge-balancing cations [48,49]. Fe_2_O_3_ is a similar case; Fe^3+^ is a glass network former in the form of a tetrahedral when Fe^3+^^/^ΣFe is >0.5; it is a glass network modifier when Fe^3+^^/^ΣFe is <0.3 [32,50,51].

**Figure 4:**
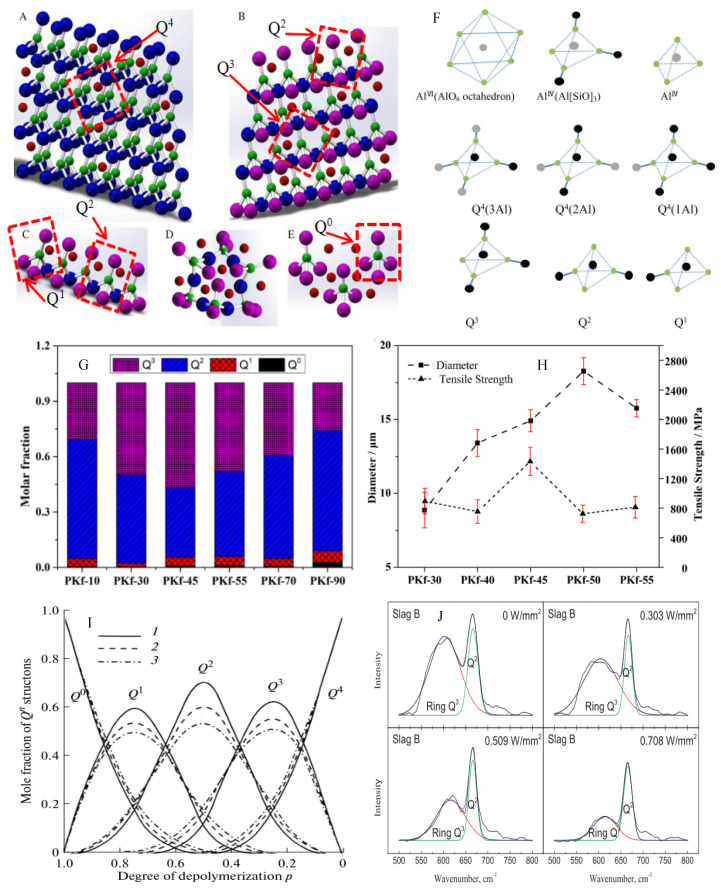
The structures of the continuous fibers. (**A**–**E**) (Xing et al., 2019) Five types of structure of the fibers. (**F**) Structure units in continuous fibers with high thermal stability (Zhang et al., 2020). (**G**) The structural composition of the fibers varies with its composition (Liu et al., 2021). (**H**) The diameter and tensile strength of the fibers varies with its composition (Liu et al., 2021). (**I**) The structural composition of melt varies with the degree of depolymerization (Polyakow et al., 2010). (**J**) Raman spectra of fluorine-bearing slag melt under ultrasound field (Min et al., 2019).

Ma et al. [32] found that continuous fiber contains five type of structures: tectosilicate, phyllosilicate, inosilicate, ring silicate, nesosilicate. The first two structures have an excellent improvement effect on fiber strength due to their structural characteristics. On the basis of the experiment results, we analyzed their structural characteristics in detail. Tectosilicate consists of a lot of Q^4^ and a little network modifier, exhibiting three-dimensional structure and a stiff network structure (Figure 4A). Phyllosilicates composed of Q^2^ and Q^3^ and a little M^2+^ and M^+^ disperse in voids of structure (Figure 4B). In contrast, the melt consisting of phyllosilicate can more easily be made into fiber because the structure is softer and can easily be drawn under the effect of force. Figure 4C indicates inosilicate consisting of Q^1^ and Q^2^. Q^2^ and Q^0^ constitute a ring silicate (Figure 4D) and nesosilicate (Figure 4E), respectively. These three structures without Q^3^ and Q^4^ cannot easily form fiber products. In conclusion, melt containing a large number of Q^2^ and Q^3^ structures benefits fiber-forming. This was the first important study result.

The second important finding was that the continuous fiber produced from fly ash and magnesium slag contained eight Si structures and three Al structures (Figure 4F) [8]. Six Si structures were Q^4^ (3Al), Q^4^ (2Al), Q^4^ (1Al), Q^4^, Q^3^ and Q^2^. They all participated in the network construction. Q^1^ was located at the edge of the network and was also a part. Q^0^ was an “isolated tetrahedron”. In three Al structures, only Al^IV^ (Al[SiO]_3_ ) was a part of the network structure. The other two structures (Al^IV^(AlO_6_ octahedron) and Al^IV^), similar to Q^0^, not only were not involved in the composition of the glassy network but also consumed M^2+^ and M^+^ ions for achieving a charge balance of O atoms. That indicated these structures made nearly no contribution to the strength of fibers. These study results indicate that not all Al can participate in the glassy network even if they meet the requirement of Al_2_O_3_ < MO + M_2_O (molar ratio). This finding contradicts previous ideas [7].

The third important finding was that Al^IV^ (AlO_6_ octahedron) can be destroyed and transformed into Al^IV^ in the vitreous melt by increasing the temperature; however, it requires too prolonged a time. Only Al^IV^ (Al[SiO]_3_) was conducive to the improvement of fiber strength and thermal stability [8].

The fourth important finding was that when the fiber was mainly composed of Q^3^ and Q^2^, and Q^3^ content is significantly more than Q^2^, in this case, the tensile strength was the highest (see PKf-45 in Figure 4G,H) [36]. This result was consistent with the theoretical analysis, because this structure combination was more likely to form a network structure that was easy to be drawn.

The fifth important finding was that adjusting the degree of depolymerization p (p = NBO/T, here T is Si or Al located in the center site of tetrahedron) in the range of 0.2 to 0.5 and controlling temperature in the near 1200 °C range can make the melt mainly contain a Q^2^ and Q^3^ structure, meanwhile keeping Q^3^ content more than Q^2^ (Figure 4I) [52]. This finding offers a way to control the melt structure, which potentially prepares the melt structure we want to obtain.

The sixth key finding was that ultrasound could quickly break Si-O bonds and increase p while decreasing the viscosity of the melt. Once the ultrasound stopped, the broken lower polymerized units reconnected to form higher polymerized units gradually (Figure 4J), the p and the viscosity reversed to their natural state of the silicate melts [53]. This indicates a way to quickly homogenize the melt and eliminate the AlO_6_ structure because the Al-O bond energy (280 kj/mol) in AlO_6_ structure was much less than the Al-O bond (422 kj/mol) and Si-O bond (443 kj/mol) in a tetrahedron structure [54,55]. Although it has not been explicitly reported, we believe that this finding suggests that ultrasonic action at high temperatures can also lead to rapid melting of solid waste blends and homogenizing. It is expected that the application of this technology in production will significantly shorten homogenization time, reduce energy consumption and carbon emissions.

Therefore, to produce high-performance continuous fibers, the melt should contain as much Q^3^ and Q^2^ structure as possible, when the Q^3^ content is more than the Q^2^ content. In relation to Al structure, we should avoid the AlO_6_ octahedron and let it transfer into Al^IV^ structure as Al[SiO]_3_.

### 3.4. Viscosity of Melts

**Figure 5:**
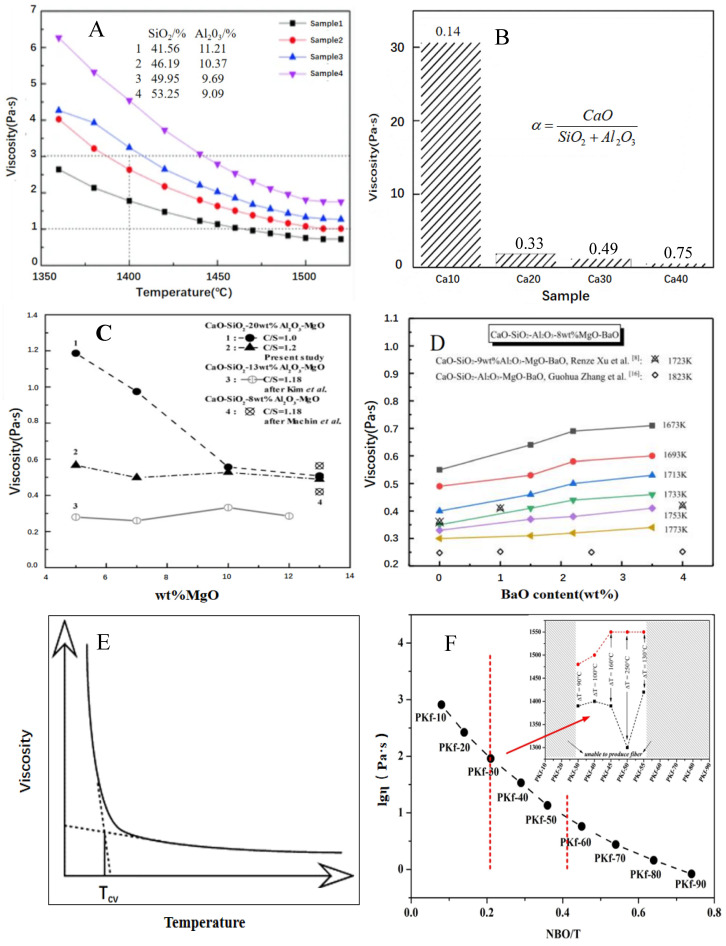
(**A**), Viscosity–temperature curves of melts with different SiO_2_ and Al_2_O_3_ content (Wu et al., 2020). (**B**) The viscosity of liquid phase of synthetic ash at 1350 °C with different α value (CaO/(SiO_2_ + Al_2_O_3_) mass ratio) (He et al., 2019). (**C**) Effect of MgO content on the viscosity of molten slag (Kim et al., 2010). (**D**) Effect of BaO content on the viscosity of molten slag (Zhang et al., 2013). (**E**) Illustration of the concept of T_cv_ or temperature of critical viscosity (Vargas et al., 2001). (**F**) Plots between the viscosity and depolymerization degree of the melts with proper fiber-forming temperature range (Liu et al., 2021).

The viscosity of melt depends on its structure and temperature. The structure of melt is related to the chemical composition and temperature. Traditional opinion states that acid oxides such as SiO_2_ and Al_2_O_3_ increase the viscosity of melt by the forming the network. Figure 5A indicates the viscosity of melt always increased as the total content of SiO_2_ and Al_2_O_3_ increased [11]. However, the network structure could be depolymerized at a high enough temperature. Therefore, the viscosity always decreased with the increasing temperature [18]. The basic oxides such as CaO, MgO, BaO, K_2_O, and Na_2_O also could break the Si-O bond and Al-O bond at high temperatures and accelerated the deploymerization of the network, which resulted in the viscosity drop with their content increasing. Figure 5B shows the viscosity obviously decreased as the α value (CaO/(SiO_2_ + Al_2_O_3_) increased when the α value was ≤0.33, then slowly dropped until several Pa.s when the α value was ≥0.33 [56]. When the MgO content increased, the viscosity change trend exhibited was obviously different depending on the CaO/SiO_2_ molar ratio (C/S). If the C/S value was 1.0, the viscosity decreased. If the C/S values were 1.18 and 2.0, the viscosity showed nearly no change (Figure 5C) [57]. BaO as a basic oxide could provide free oxygen ions (O^2−^) to depolymerize the network structure and decrease the viscosity. However, the experiment result showed the addition of BaO could slightly increase the viscosity of the melt (Figure 5D) [58]. This result was truly anomalous. An explanation was that it needed cations to charge-compensate when Al^3+^ integrated into the Si^2+^ structure, which could form a complex aluminate–silicate structure and increase the viscosity of the melt [58,59]. In this case, CaO and MgO also needed cations to charge-compensate. But their effects on viscosity were obviously different. The exact reason needs to be further studied.

Fe_x_O_y_ have a complicated effect on viscosity, either increasing or decreasing viscosity, which is determined by the composition of melts [60]. Fe^2+^ content increased at reducing atmosphere, which caused the decrease in viscosity [61]. The spinel would occur and increased viscosity with Fe_2_O_3_/(SiO_2_ + Al_2_O_3_) value increasing [34,62,63], which indicated Fe^3+^ caused crystalline occurrence in melts and made Newtonian fluid melt transfer into non-Newtonian fluid melt. That did not benefit the fiber-forming process, therefore it should be iron-free or iron content should be as little as possible, which is in line with the previous discussions.

Figure 5E shows the critical temperature (T_cv_) in the viscosity–temperature curve of the melt [24]. This was the cut-off point from Newtonian to Non-Newtonian fluid [25,64,65,66,67]. The melt was in a glassy state and a Newtonian fluid when temperature was above T_cv_. In this case, the viscosity slowly increased with the temperature dropped due to the increase in the polymerization degree of the melt. When temperature was below T_cv_, the melt was non-Newtonian fluid because micro-crystalline solids had occurred. The viscosity drastically dropped as the temperature decreased. For the melt assigned to Newtonian fluid, the following Arrhenius-type equation was applied to express the temperature-dependence of the viscosity η:

$$\ln\;\eta =\ln\;A+\left(\frac{En}{RT}\right)$$

Here, η was the viscosity of melt, (Pa.s); A was pre-exponent factor, *En* was the apparent activation energy of the melt, (J/mol); *R* was the gas constant, (8.314 J/mol.K); *T* was the absolution temperature, (K) [59].

Owing to this dependence relationship between the *T* and η of melt, in practical production, the T_ff_ range of melt will always be determined by the suitable viscosity range.

For the production of wool fibers, a suitable range is from 1.0 to 3.0 Pa.s; the general temperature range was 1450–1550 °C according to the accumulation of experience [6,68,69]. For the production of continuous glass fibers, the suitable viscosity range is from 31.7 to 100 Pa.s according to the requirement of glass fibers [70]. For the continuous fibers from solid waste, the temperature range was from 1200 to 1410 °C as noted in the previous summary. In practice, there were some differences in these technology parameter ranges (viscosity and temperature) due to the difference in the chemical composition of various solid waste blends. For example, some continuous fibers from pyroxene and K-feldspar had a production viscosity range of lgµ: 0.9–1.9 (equivalent to a viscosity of 8–80 Pa.s) (Figure 5F); fibers were not drawn successfully beyond this range [36].

## 4. The Relationship between Winding Speed, Diameter, Mk, and Tensile Strength of the Fibers Produced from Solid Wastes

The main technology parameters in the production of fibers include winding speed, viscosity and temperature. In theory, the relationship between diameter of fiber and winding speed can be expressed by the following equation [71,72]:

$$\pi {\left(\frac{d_{1}}{2}\right)}^{2}=\pi {\left(\frac{d_{2}}{2}\right)}^{2},\;{\left(\frac{d_{1}}{d_{2}}\right)}^{2}=\frac{L_{2}}{L_{1}}$$

**Figure 6:**
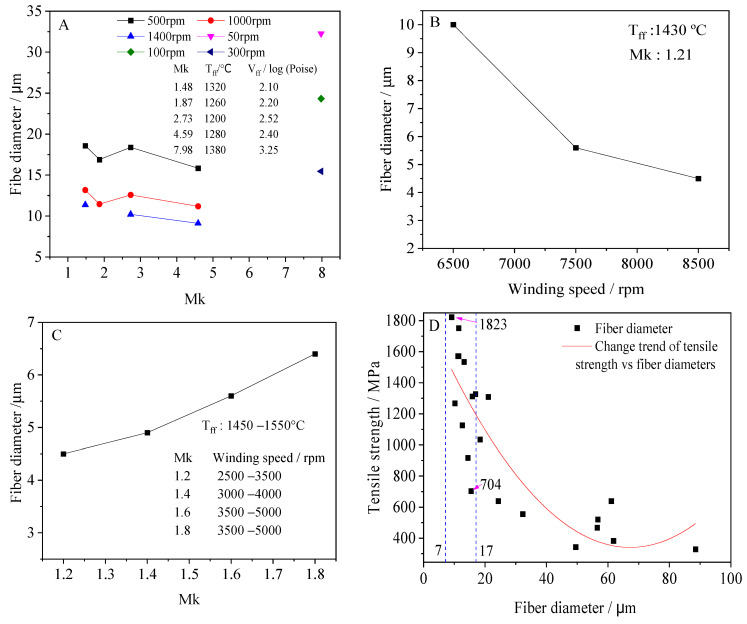
The relationship between tensile strength, diameter, Mk, and winding speed of fiber produced from solid wastes. (**A**) The relationship of Mk and the diameter of continuous fibers. (**B**) The relationship of winding speed and diameter of mineral fibers. (**C**) The relationship of Mk and the diameter of mineral fibers. (**D**) The relationship of tensile strength and diameter of continuous fibers. The above data in figures from (Kim et al., 2018; Ko et al., 2021; Li et al., 2018; Chan et al., 2017).

Here, *L* and *d* were the winding speed and fiber diameter, respectively. It could be applied only if the same melt was at the same temperature. As shown in Figure 6A,B, the fiber diameter always decreased as the winding speed increased for both continuous fibers and mineral fibers. At the same winding speed, the diameter of continuous fiber appeared to bear no obvious relationship with the Mk and the V_ff_ when the winding speed was low. For example, at the winding speed of 500 rpm, the diameter of the fibers at the V_ff_ of logη (poise) = 2.10 was slightly greater than the fibers at the V_ff_ of logη (poise) = 2.20 and logη (poise) = 2.40, while the diameter of the fibers at the V_ff_ of logη (poise) = 2.52 was a similar case. However, when the winding speed increased up to 1400 rpm, regardless of the V_ff_, the fiber diameter slowly decreased with an increase of the Mk in the range of 1.48 to 4.59 (Figure 6A). If the Mk was too high, the fiber could not be formed at high wind speed. At least until now, it has been thought. For example, the fiber with a Mk of 7.98 could only be successfully drawn at a low winding speed of ≤300 rpm [13]. However, for mineral fiber, although the winding speed increased, its diameter always increased with the increase of the Mk (Figure 6C). This difference between the two types of fibers may result from different fiber-forming methods and equipment. Figure 6D shows the scatter chart of the diameter and tensile strength of continuous fiber produced from solid wastes. In spite of the fact that fibers were obtained at different conditions, the statistics data show that continuous fibers with high strength were always fine fibers, very much like fiberglass and basalt fiber, considering common basalt fibers have a diameter range of 7 to 17 µm and the reported tensile strength of a single basalt fiber is 3000 to 4840 Mpa [73]. The continuous fibers from solid wastes in the present study only have a tensile strength from 704 to 1823 Mpa and a diameter range of 9.11 to16.89 (Figure 6D); there is still a significant difference between them.

## 5. Conclusions and Outlook

The production of solid waste fibers is a good way to recycle solid waste resources. How to effectively use local solid waste resources to produce fiber, especially for continuous fibers, is an important research topic in this field. In this review, the key factors affecting the properties of fibers and melts were discussed and summarized. The specific conclusions are as follows,

(1) For fiber production, matching of solid wastes containing enough total content of SiO_2_ and Al_2_O_3_, and a suitable amount of MgO and CaO was beneficial to the structure control of the melt.
(2) The study found that the melt consisted of Q_2_ and Q_3_, and Q_3_ content more than Q_2_, and was more suitable for the production of fibers and production performance improvement. Thus, melt structure can be obtained by controlling the degree of depolymerization and suitable temperature range.
(3) Further study showed that the viscosity of the melt could be effectively controlled by regulating its chemical composition, especially the content of the network formers.
(4) The optimum technology parameters for fiber production also were found. The new ultrasonic technology could rapidly shorten the homogenization time, save energy costs and reduce carbon emissions. The practical application of these findings in production will promote the development of the solid waste fiber industry while reducing energy consumption and carbon emissions.

## Figures and Tables

**Table 1:** Chemical composition, production method, mean diameter, tensile strength of the mineral fibers from solid wastes in recent 12 years.

Fiber Raw	SiO_2_	Al_2_O_3_	CaO	MgO	K_2_O	Na_2_O	Fe_x_O_y_	TiO_2_	B_2_O_3_	Method	T_ff_	Diameter*	Strength	Mk	Ref.
	Wt%										°C	μm	MPa		
Fa + Bo	35.60	11.90	24.61	1.84	0.73	4.27	9.73	--	--	Blowing	1600	0.5–5.5	--	1.80	[17]
BQFB	35.39	15.74	34.93	7.44	--	--	1.77	--	--	Centrifugal	1430	4.5	--	1.21	[23]
MSS	32.74	10.0	24.86	3.80	--	--	--	--	--	Centrifugal	1350	5.1	--	1.49	[42]
BFS	36.10	26.70	25.30	6.20	0.60	0.40	1.50	1.40	--	Centrifugal	--	7.0	2579	1.99	[43]
CSCD	54.32	13.58	14.01	8.13	--	--	6.05	--	--	Centrifugal	1600	11.43	1806	3.07	[44]
CHCS	47.68	13.96	33.68	4.86	--	--	--	--	--	Centrifugal	1450	≤5.0	--	1.60	[19]
BFS + quartz 1	41.56	11.21	38.99	4.86	--	--	--	--	--	Centrifugal	1450–1550	4.5	--	1.20	[18]
BFS + quartz 2	46.19	10.37	35.75	4.46	--	--	--	--	--	Centrifugal	1450–1550	4.9	--	1.41	[18]
BFS + quartz 3	49.95	9.69	33.10	4.13	--	--	--	--	--	Centrifugal	1450–1550	5.6	--	1.60	[18]
BFS + quartz 4	53.25	9.09	30.79	3.84	--	--	--	--	--	Centrifugal	1500–1600	6.4	--	1.80	[18]
Fes + B_2_O_3_ 1	49.28	5.35	3.51	29.36	0.64	0.31	6.68	0.13	2.93	Blowing	1500	5.6	1724	1.66	[20]
Fes + B_2_O_3_ 2	48.49	5.23	3.26	28.94	0.62	0.30	6.53	0.13	4.67	Blowing	1500	4.7	1775	1.67	[20]
Fes + B_2_O_3_3	47.41	5.06	3.14	28.38	0.60	0.29	6.65	0.12	6.58	Blowing	1500	4.3	1810	1.66	[20]
Fes + B_2_O_3_ 4	46.60	4.98	3.17	27.79	0.60	0.29	6.51	0.12	8.17	Blowing	1500	3.4	2114	1.67	[20]

Note: Fa + Bo: fly ash + bottom ash; BQFB, blast furnace slag + quartz + fly ash + basalt; MSS, manganese slag + silicon slag; BFS, blast furnace slag; CSCD, copper slag + coal gangue + dolomite; CHCS, chromium slag + coal slag; Fes: ferronickel slag; Diameter*: mean diameter.

**Table 2:** Chemical composition, production method, diameter, tensile strength of the continuous fibers from solid wastes.

Fiber	SiO_2_	Al_2_O_3_	CaO	MgO	K_2_O	Na_2_O	Fe_x_O_y_	TiO_2_	T_ff_	Diameter	Strength	Speed	Mk	Ref.
	Wt%								°C	μm	MPa			
F45	53.4	12.58	21.17	--	--	--	9.70	--	1330	35.0	420	50 m/s	3.12	[45]
GWRF	45.4	12.40	10.20	11.2	1.00	1.90	15.4	2.40	1230	61.1	639	1.3 m/s	2.70	[10]
Faf1	55.43	19.59	6.57	2.76	1.92	2.34	5.89	0.74	1380	17.0	704	300 rpm	8.04	[12]
Faf2	55.84	13.67	17.31	6.36	1.54	2.75	5.15	0.65	1260	13.0	1753	1000 rpm	2.94	[12]
Faf3	38.75	13.39	15.90	6.02	1.29	1.30	4.57	0.62	1320	11.58	1650	1400 rpm	2.38	[12]
FMPM	47.7	18.8	15.0	4.60	2.47	1.32	2.49	--	1410	14.04	903	5 m/s	3.39	[8]
FMM	38.6	16.1	27.1	7.15	0.85	0.37	3.15	--	1320	25.75	539	5 m/s	1.60	[8]
VF1	52.5	14.3	21.1	3.40	0.30	1.40	0.40	--	1200	10.20	1268	1400 rpm	2.73	[13]
VF3	48.4	19.1	11.3	3.40	2.30	1.80	2.50	0.60	1280	9.11	1823	1400 rpm	4.59	[13]
VF6	38.9	13.4	25.4	9.90	1.30	1.30	4.60	0.60	1320	11.37	1571	1400 rpm	1.48	[13]

Note: GWRF, gold tailings: waste limestone: red mud: ferronickel slag = 25:20:15:40 (mass ratio); FMM, fly ash: magnesium slag =108:100 mass ratio); FMPM: fly ash: magnesium slag: potassium feldspar: soda feldspar = 4:2:1:1 (mass ratio); Fa45, Fly ash: agents (SiO_2_ + CaCO_3_ + MgCO_3_) = 45:55 (mass ratio); Faf1, fly ash: basalt:anorthite:feldspar = 6:1:1:2, (300 rpm) Faf2, fly ash: dolomite:calcite:frit = 6:2:1:1 (Burr:22%), (1000 rpm); Faf3, fly ash: dolomite:calcite:feldspar = 6:2:1:1, (1400 rpm).

## Data Availability

Not applicable.
